# High‐yield recombinant bacterial expression of ^13^C‐, ^15^N‐labeled, serine‐16 phosphorylated, murine amelogenin using a modified third generation genetic code expansion protocol

**DOI:** 10.1002/pro.4560

**Published:** 2023-02-01

**Authors:** Garry W. Buchko, Mowei Zhou, Cat Hoang Vesely, Jinhui Tao, Wendy J. Shaw, Ryan A. Mehl, Richard B. Cooley

**Affiliations:** ^1^ Earth and Biological Sciences Directorate Pacific Northwest National Laboratory Richland Washington USA; ^2^ School of Molecular Biosciences Washington State University Pullman Washington USA; ^3^ Department of Biochemistry and Biophysics Oregon State University Corvallis Oregon USA; ^4^ Physical and Computational Sciences Directorate Pacific Northwest National Laboratory Richland Washington USA

**Keywords:** ^31^P‐NMR, amelogenesis, amelogenin, biomineralization, isotope labeling, nanospheres, phosphoproteins, posttranslational modifications, recombinant protein expression

## Abstract

Amelogenin constitutes ~90% of the enamel matrix in the secretory stage of amelogenesis, a still poorly understood process that results in the formation of the hardest and most mineralized tissue in vertebrates—enamel. Most biophysical research with amelogenin uses recombinant protein expressed in *Escherichia coli*. In addition to providing copious amounts of protein, recombinant expression allows ^13^C‐ and ^15^N‐labeling for detailed structural studies using NMR spectroscopy. However, native amelogenin is phosphorylated at one position, Ser‐16 in murine amelogenin, and there is mounting evidence that Ser‐16 phosphorylation is important. Using a modified genetic code expansion protocol we have expressed and purified uniformly ^13^C‐, ^15^N‐labeled murine amelogenin (pS16M179) with ~95% of the protein being correctly phosphorylated. Homogeneous phosphorylation was achieved using commercially available, enriched, ^13^C‐, ^15^N‐labeled media, and protein expression was induced with isopropyl β‐D‐1‐thiogalactopyranoside at 310 K. Phosphoserine incorporation was verified from one‐dimensional ^31^P NMR spectra, comparison of ^1^H‐^15^N HSQC spectra, Phos‐tag SDS PAGE, and mass spectrometry. Phosphorus‐31 NMR spectra for pS16M179 under conditions known to trigger amelogenin self‐assembly into nanospheres confirm nanosphere models with buried N‐termini. Lambda phosphatase treatment of these nanospheres results in the dephosphorylation of pS16M179, confirming that smaller oligomers and monomers with exposed N‐termini are in equilibrium with nanospheres. Such ^13^C‐, ^15^N‐labeling of amelogenin with accurately encoded phosphoserine incorporation will accelerate biomineralization research to understand amelogenesis and stimulate the expanded use of genetic code expansion protocols to introduce phosphorylated amino acids into proteins.

## 1 INTRODUCTION

Protein phosphorylation is the most common posttranslational modification cells use to regulate protein function (Pawson & Scott, 2005; Ubersax & Ferrell, 2007). Included among the basic cellular processes regulated by protein phosphorylation is amelogenesis (Shin et al., 2020), an activity that results in the formation of the hardest, toughest, and most mineralized tissue in vertebrates––enamel (Simmer & Fincham, 1995; Ten Cate, 1994). Enamel is composed of narrow crystals of carbonated hydroxyapatite (Ca_10_(PO_4_)_6_(OH)_2_) and its extraordinary mechanical properties arise from its weaving into an unique, closely packed, lattice architecture (Daculsi et al., 1984; Hunter, 1996; Margolis et al., 2006). Amelogenesis occurs in specialized epithelial cells called ameloblasts and is regulated by the proteins amelogenin, ameloblastin, enamelin, and amelotin. Of these proteins, the dominant protein (>90%) at the secretory stage of amelogenesis is amelogenin (Margolis et al., 2006; Simmer & Fincham, 1995). Genetically modified and knock‐out mouse models show amelogenin is a key component for proper enamel formation (Gibson et al., 2001; Hu et al., 2016). This importance is corroborated by human genetic studies showing mutations to the amelogenin gene AMELX is a leading cause of *amelogenesis imperfecta* (Ravassipour et al., 2000; Witkop Jr. et al., 1973), a heterogenous group of hereditary conditions that affect the quantity and quality of enamel (Smith et al., 2017). The AMELX gene product contains only one posttranslational modification, the phosphorylation of a single serine residue near the N‐terminal which in human, mice, and pig amelogenin is Ser‐16 (pSer‐16).

Analysis of the primary amino acid sequence of amelogenin from humans and other vertebrate tetrapods shows that the sequence is highly homologous across species (Toyosawa et al., 1998) and can be divided into three domains: an N‐terminal, hydrophilic tyrosine‐rich region (a.k.a. TRAP), a hydrophobic central region heavily populated with histidine, glutamine, and proline residues, and a hydrophilic mineral binding C‐terminal region (Margolis et al., 2006; Shaw et al., 2020). The N‐ and C‐terminal domains are highly conserved across species with small species‐dependent variations in the length of amelogenin due largely to differences in the length of the central domain. Structural studies suggest amelogenin is an intrinsically disordered protein especially in acidic aqueous solution under dilute conditions where it is primarily monomeric (Buchko, Tarasevich, et al., 2008; Delak et al., 2009). Perhaps due to the histidine residues in the central domain (Bromley et al., 2011), as the solution pH is increased amelogenin self‐assembles into different quaternary structures in a dynamic, step‐wise fashion (Bromley et al., 2011; Lakshminarayanan et al., 2007; Moradian‐Oldak et al., 1994; Moradian‐Oldak et al., 1998). Starting at low pH (<~3.5) ameloginin is primarily monomeric (Buchko, Tarasevich, et al., 2008; Delak et al., 2009). As physiological pHs are approached (pH ~6.6) amelogenin forms oligomers of increasing size up to a maximum average size of about eight monomers (Du et al., 2005; Fang et al., 2011). At pH values above ~7.2, nanospheres composed of 20–100 monomers form (Bromley et al., 2011; Du et al., 2005) and, under the right conditions, nanosphere chains (Wiedemann‐Bidlack et al., 2007) and nanoribbons (Carneiro et al., 2016; He et al., 2011). Nanospheres have been observed both *in vitro* (Fincham et al., 1994) and *in vivo* (Fincham et al., 1995), and appear to be essential for proper enamel formation (Paine et al., 2000). In addition to pH, the equilibrium between various quaternary structures is influenced by other factors including the amelogenin concentration, the presence of amelogenin degradation products, and the properties of the solution such as ionic strength, solutes, and temperature (Engelberth et al., 2018; Moradian‐Oldak et al., 1998; Shaw et al., 2020).

While there is little doubt that amelogenin plays a critical role in amelogenesis, how this ~20 kDa, intrinsically disordered protein controls hydroxyapatite (HAP) crystal growth at the molecular level is still not well understood. A contributing factor toward this knowledge gap may be because a significant amount of the biophysical research on amelogenin has been conducted using recombinant protein expressed in *Escherichia coli*. While recombinant methods have been successful in providing copious amounts of protein for various biophysical studies and ^13^C‐ and ^15^N‐labeled protein for detailed structural studies using NMR spectroscopy (Buchko et al., 2022; Buchko, Bekhazi, et al., 2008; Buchko, Tarasevich, et al., 2008; Delak et al., 2009), recombinant amelogenin expressed in *E. coli* lacks a phosphoserine. A few scattered experiments using native phosphorylated amelogenin (Fang et al., 2013; Wiedemann‐Bidlack et al., 2011), peptides of native amelogenin digestion products (Kwak et al., 2009), or the amelogenin splice‐variant LRAP chemically synthesized with pSer‐16 (Lu et al., 2013; Yamazaki et al., 2017) have hinted that amelogenin phosphorylation may affect the physical properties of the protein and play a role in regulating enamel formation. Recently, Margolis et al. provided some of the strongest *in vivo* evidence to date for the importance of amelogenin phosphorylation using a hemizygous knock‐in mouse model with a Ser‐16 to Ala substitution in the AMELX gene (Shin et al., 2020). The enamel of such genetically engineered mice was defective, lacking enamel rods and exhibiting hypoplasia and numerous surface defects. To accelerate the biomineralization community's efforts to more fully explore the biophysical effects of amelogenin phosphorylation, we describe the application of the latest generation of genetic code expansion technology (Zhu et al., 2019) for obtaining milligram quantities of ^13^C‐ and ^15^N‐labeled, Ser‐16 phosphorylated, murine amelogenin (pS16M179) using recombinant methods in *E. coli*. The advantage of illustrating this method with ^13^C‐ and ^15^N‐labeled protein is because the NMR chemical shifts for non‐phosphorylated recombinant murine amelogenin (M179, a 179‐residue protein after N‐terminal methionine removal) are known (Buchko, Bekhazi, et al., 2008; Buchko, Tarasevich, et al., 2008), and consequently, ^13^C‐ and ^15^N‐labelling allows us to readily verify the presence of Ser‐16 phosphorylation, the extent of Ser‐16 phosphorylation, and the stability of Ser‐16 phosphorylation. Note that genetic code expansion technologies that encode for phosphoserine use engineered *E. coli* expression hosts that lack the *serB* gene. *SerB* encodes for a serine phosphatase that converts free phosphoserine amino acids into serine as part of the serine biosynthesis pathway. By deleting *serB*, free pSer builds up inside the cells and feeds the engineered pSer genetic code expansion system. Because these *serB* ^−^ *E. coli* expression hosts are serine auxotrophs, classical ^13^C‐ and ^15^N‐labeling protocols using minimal media are ineffective (Zhu et al., 2019). Instead, it is necessary to use enriched, ^13^C‐ and ^15^N‐labeled media, such as Celtone or BioExpress (Vesely et al., 2022).

## 2 RESULTS AND DISCUSSION

### 2.1 Expression of phosphorylated pS16M179

To genetically encode phosphoserine at amber stop codons in *E. coli* during translation, we employed a pSer genetic code expansion machinery system previously developed by Chin and colleagues (Rogerson et al., 2015). These expression systems rely on the use of expression hosts that lack the *serB* gene as a means to build up free phosphoserine amino acid inside the cell, which serves to feed the genetic code expansion machinery. Because *serB* is critical for serine biosynthesis, these *E. coli* expression hosts are serine auxotrophs and cannot grow in traditional, isotopically enriched minimal media. However, we observed that normal growth of these serine auxotrophs could be restored by supplementing minimal media with Celtone, an algal extract which can be commercially purchased enriched with various isotopes, including ^15^N and ^13^C (Vesely et al., 2022). Using such media we recently optimized a high‐density culturing method using BL21(DE3) Δ*serB* as an expression host, which enabled for the first time, high yields of homogeneously phosphorylated, isotopically labeled proteins. However, our attempts to express phosphorylated amelogenin in high yields with this strain and these methods were not successful (data not shown).

We hypothesized that using a strain of *E. coli* called B95(DE3) Δ*A* Δ*fabR* Δ*serB*, which lacks Release Factor‐1 (RF1, the protein responsible for terminating translation at amber codons) could improve incorporation by removing competition between the amber suppressing, pSer‐amino acylated tRNA and RF1. The absence of RF1, however, opens the door for endogenous tRNAs (e.g Gln‐tRNA) to suppress UAG codons via wobble‐base pairing (Beyer et al., 2020; Zhu et al., 2019), a phenomenon referred to as near‐cognate suppression. Therefore, checking the accuracy of encoding when using RF1 deficient expression hosts is particularly important to ensure the GCE machinery can effectively outcompete endogenous near‐cognate suppressor tRNAs. Indeed, we previously demonstrated accurate encoding of pSer when using rich auto‐induction media with the B95(DE3) Δ*A* Δ*fabR* Δ*serB* expression host (Zhu et al., 2019).

We therefore tested whether this cell line, B95(DE3) Δ*A* Δ*fabR* Δ*serB*, could be used to produce isotopically enriched, phosphorylated proteins using our high‐density culturing methods previously optimized with BL21(DE3) Δ*serB*. Similar or improved yields of the model protein, super‐folder green fluorescent protein with an amber (TAG) stop codon for phosphoserine at residue position 150 (sfGFP‐150TAG) (Zhu et al., 2019) were obtaining using Phos‐tag gel electrophoresis to assess the proportion of purified protein that was phosphorylated (Figure S1). Phos‐tag gels contain a di‐nuclear metal complex (1,3‐bis[bis‐(pyridine‐2‐ylmethyl)amino]propan‐2‐olato dizinc(II)) copolymerized in an acrylamide matrix with affinity for phosphate groups that retards phosphoprotein migration in the gel during electrophoresis (Kinoshita et al., 2006). The method is sensitive enough to separate a protein by the number of sites phosphorylated (Kinoshita et al., 2006; Zhu et al., 2019). Unfortunately, an unacceptable proportion of the purified protein (up to 90%) was not phosphorylated using these high‐density expression methods due to near‐cognate suppression. Through rounds of further expression optimization, we found that reverting back to a low‐density culturing method and expressions at elevated temperatures (298–310 K) notably improved the faithful encoding of pSer into sfGFP‐150TAG (up to 90%) using the B95(DE3) Δ*A* Δ*fabR* Δ*serB* expression host (Figure S1).

**FIGURE 1:**
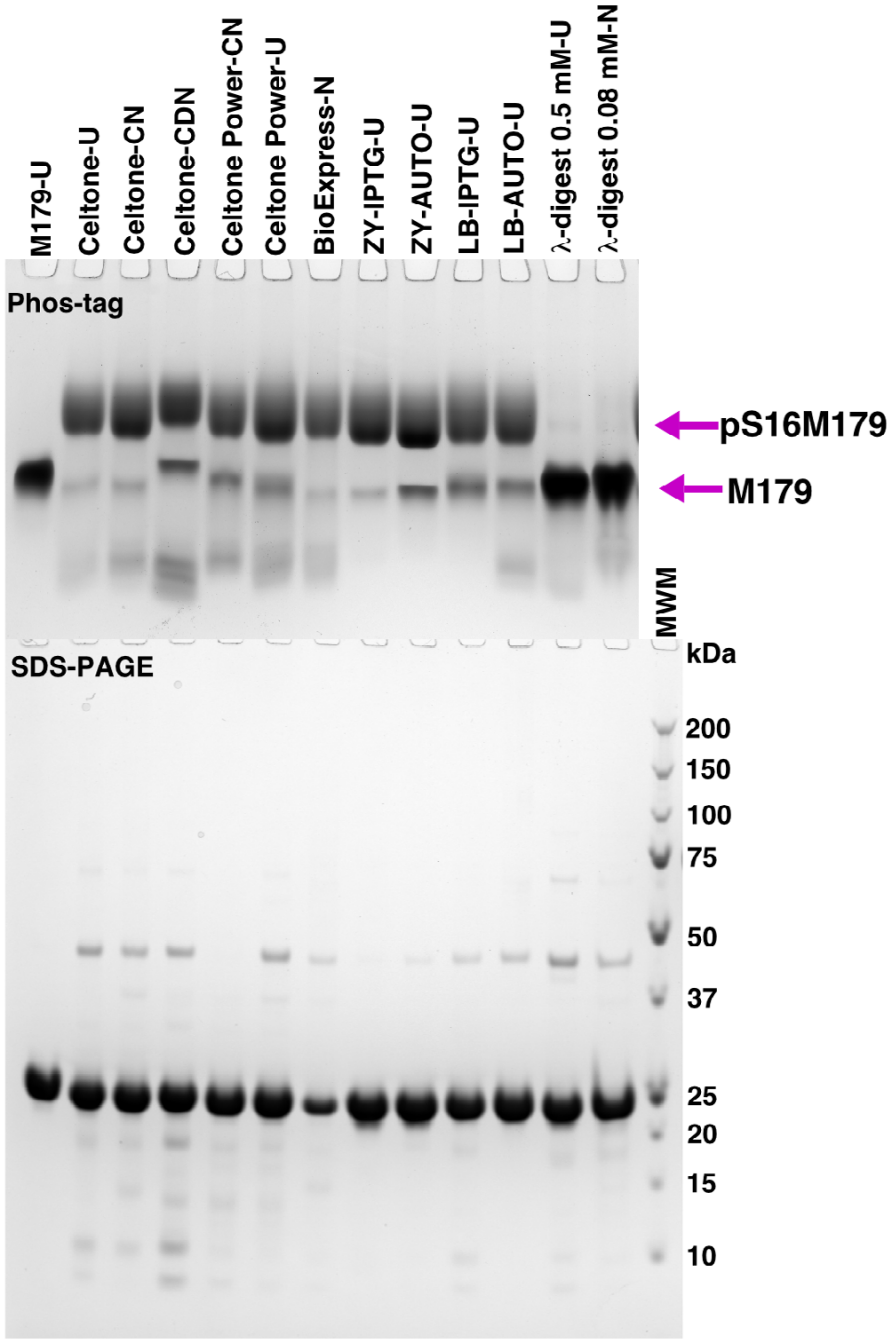
Coomassie‐stained Phos‐tag (top) and SDS‐PAGE (bottom) gels for M179 and pS16M179 expressed in B21(DE3) and B95(DE3) *E. coli*, respectively, using a suite of media. The last two lanes are pS16M179 treated with lambda phosphorylase (λ) at two different concentrations (0.5 and 0.08 mM). Approximately 5 μg of protein was loaded into each lane (except the SDS‐PAGE lane for BioExpress‐N). Protein expression was induced with IPTG in the Celtone and BioExpress media. Labeling code: U = unlabeled (natural abundance); C = carbon‐13; N = nitrogen‐15; D = deuterium

Guided by these improvements using sfGFP as a model protein, we asked whether the B95(DE3) Δ*A* Δ*fabR* Δ*serB* strain could be used to express phosphorylated amelogenin and tested a variety of culturing and expression conditions. Figure 1 shows a Phos‐tag gel for purified pS16M179 obtained from ^13^C‐, ^15^N‐labeled Celtone complete and other media, and Table 1 summarizes the approximate yields of amelogenin using these media. A number of general conclusions can be made from an inspection of the gel. First, un‐phosphorylated M179 clearly migrates faster than pS16M179, confirming the phosphorylation of the protein. Second, relative to M179, only one major slower migrating band is detected indicating that the phosphorylation is confined to one site. Third, based on a visual inspection of the gel, the level of pSer incorporation is high regardless of the media used during protein expression. Fourth, IPTG induction in ZY‐media results in moderately lower levels of misincorporation for pSer than autoinduction in ZY‐media, however, the yields of amelogenin are about 3× greater using autoinduction. Fifth, the level of misincorporation was greatest in the deuterated Celtone complete media compared to the media using 100% H_2_O and there was a greater number of faster migrating impurities in the sample perhaps due to the slower growth rate of *E. coli* grown in D_2_O (Opitz et al., 2019). For murine amelogenin at least, rapid growth appeared essential for the expression of pS16M179 as no protein expression was observed in ZY‐media at 293 K (Table 1).

**TABLE 1:** Approximate amelogenin yields obtained using various media and induction methods

Media	Induction	Temperature (K)	Approximate yields (mg/ml)
Celtone uniform complete	IPTG	310	10–15
Celtone ^13^C‐, ^15^N‐complete	IPTG	310	10–15
Celtone ^2^H‐, ^13^C‐, ^15^N‐complete	IPTG	310	5–10
Bioexpress ^15^N, 10x	IPTG	310	10–15
Celtone ^13^C‐, ^15^N‐powder	IPTG	310	~5
Celtone unlabeled powder	IPTG	310	~10
ZY^b^	IPTG	310	~10
ZY	AUTO	310	~30
ZY	AUTO	293	none
LB^a^	AUTO	310	~5
LB	IPTG	310	~15

^a^ LB media = 10 g tryptone, 5 g yeast extract, and 10 g NaCl per liter.

^b^ ZY media = 10 g tryptone and 5 g yeast extract per liter.

### 2.2 NMR verification of pSer‐16 incorporation into pS16M179

**FIGURE 2:**
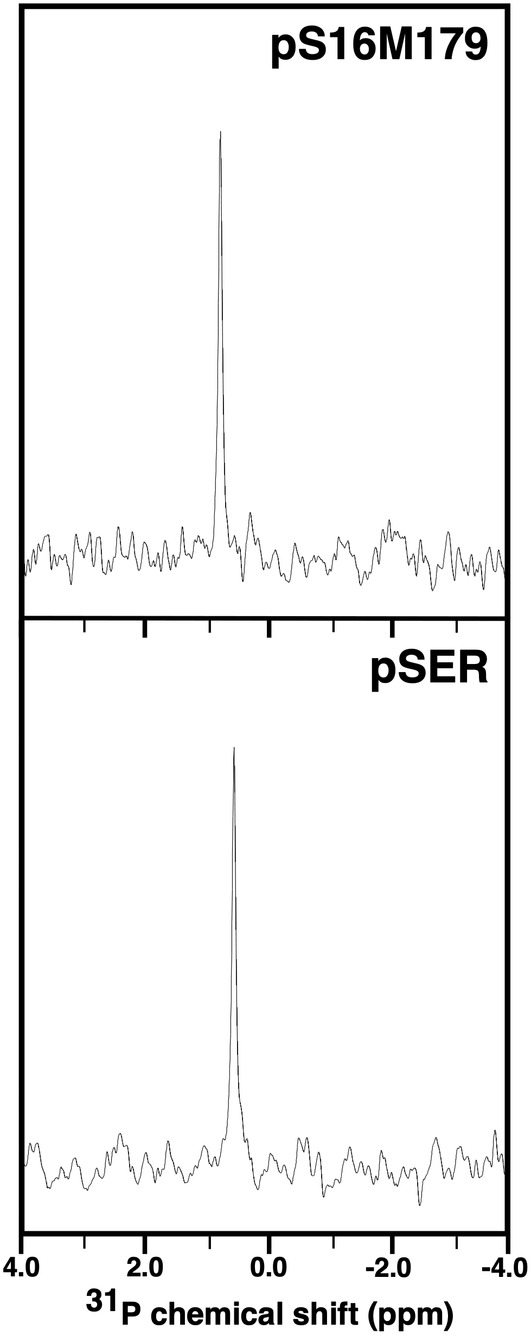
One‐dimensional ^31^P NMR spectra of 0.1 mM pS16M179 (top) and 1.0 mM pSer (bottom) collected in 2% acetic acid (pH 2.8) at 293 K and a ^1^H resonance frequency of 500 MHz. There was no change in both spectra following the incubation of both samples in their NMR tubes for over 1 month at room temperature. The ^31^P spectra were referenced to 85% phosphoric acid (0.0 ppm).

Additional indication that the genetic code expansion method for incorporating phosphoserine into M179 worked was the one‐dimensional ^31^P NMR spectrum for pS16M179 shown in Figure 2 (top). At pH 2.8 a single resonance at 0.7 ppm was observed which is near the chemical shift observed for the phosphoryl group in pSer at 0.5 ppm (bottom). As shown in Table 2, these ^31^P chemical shifts are also near the chemical shift for the phosphorylated serine observed in a number of short, random coil peptides at pH 3 (Bienkiewicz & Lumb, 1999; Hoffmann et al., 1994), suggesting this region of pS16M179 is also disordered. This is in agreement with earlier studies showing non‐phosphorylated murine and porcine amelogenin are intrinsically disordered at pH 2.8 (Buchko, Tarasevich, et al., 2008; Delak et al., 2009). Note that there were no noticeable changes to one‐dimensional ^31^P spectra of pS16M179 and pSer at pH 2.8 upon incubation of the samples in the NMR tubes at 293 K for over a month, indicating that serine phosphorylation was stable under these conditions over this time frame and, at least for amelogenin expression in *E. coli*, would not require the use of a nonhydrolyzable analog of phosphoserine (Rogerson et al., 2015).

**TABLE 2:** Select NMR chemical shifts (ppm) for phosphorylated and non‐phosphorylated Ser in M179 and pS16M179 and for the free amino acids pSer and Ser, all in 2% acetic acid, pH 2.8

	Random coil^a^	M179^b^	pS16M179	Ser^c^	pSer^c^
^13^Cα	58.1	58.9	57.5	58.6	57.1
^13^Cβ	64.1	63.6	66.2	62.6	65.9
Backbone amide ^1^H^N^	8.36	8.17	8.42	‐	‐
Backbone amide ^15^N	116.8	116.0	115.0	‐	‐
^31^P	1.2^d^	‐	0.7	‐	0.5

^a^ Serine random coil values are from the BMRB and reflect near neutral pH values.

^b^ BMRB entry 15,662 collected on an ~2 mM sample of M179 containing the N‐terminal tag MRGSHHHHHHGS‐, in 2% acetic acid, pH 2.8 (Buchko, Bekhazi, et al., 2008).

^c^ Collected on 1 mM samples under the same conditions as for pS16M179 and M179 (2% acetic acid, pH 2.8).

^d^ From Beinkiewicz & Lumb (1999) converted to phosphoric acid referencing.

**FIGURE 3:**
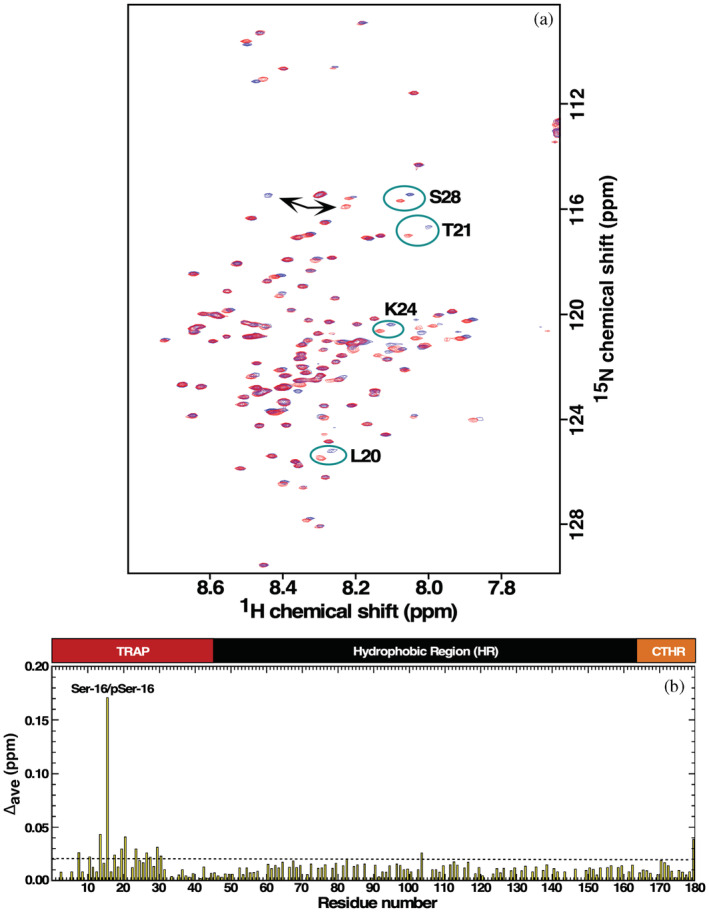
(a) Overlay of the ^1^H‐^15^N HSQC spectra for ^13^C‐,^15^N‐labeled M179 (red) upon pS16M179 (blue) collected in 2% acetic acid (pH 2.8) at 293 K. Spectra were collected on 0.3 mM samples, at a ^1^H resonance frequency of 600 MHz. (b) Plot of the average chemical shift change, Δ_ave_ ppm = {[(Δ^1^H^N^)^2^ + (Δ^15^N/5)^2^]/2}^1/2^, between the amide resonances of pS16M179 and M179 extracted from ^1^H‐^15^N HSQC spectra. The most perturbed resonance is the chemical shift difference between Ser‐16 and pSer‐16 (labeled with arrows) with mild perturbations confined to a region surrounding pSer‐16 in the N‐terminal. On top of the plot is a cartoon representation of the three regions present in amelogenin: N‐terminal tyrosine‐rich region (TRAP; red), hydrophobic region (HR; black) and C‐terminal hydrophilic region (CTHR; orange)

While the one‐dimensional ^31^P spectrum for pS16M179 showed that phosphoserine had been incorporated into M179, we sought additional quantitative information on the homogeneity of amelogenin phosphorylation. Nitrogen‐15 labeling provides a sensitive method for quantitating the extent of pSer incorporation through the collection of a “fingerprint” two‐dimensional ^1^H‐^15^N HSQC spectrum. Such fingerprint ^1^H‐^15^N HSQC spectra have been used to quickly assess the success of expressing ^15^N‐labeled M179 constructs containing point mutations (Buchko et al., 2013; Buchko & Shaw, 2015) or point deletions (Buchko et al., 2018; Tao et al., 2022) by collecting ^1^H‐^15^N HSQC spectra in 2% acetic acid (pH 2.8), a condition where amelogenin is intrinsically disordered, primarily monomeric in solution (especially at concentrations under 0.5 mM), and the amide chemical shifts have been assigned (Buchko, Bekhazi, et al., 2008; Buchko, Tarasevich, et al., 2008). Figure 3a overlays the ^1^H‐^15^N HSQC spectrum of M179 (red) and pS16M179 (blue) and shows the disappearance of the Ser‐16 amide cross peak in the pS16M179 spectrum and the appearance of a new amide cross peak in this region (black arrows). Analysis of the pS16M179 HNCACB data indicates this amide is connected to a positive and negative cross peak corresponding to ^13^C^α^ and ^13^C^β^ with chemical shifts expected for a phosphoserine residue in a disordered protein (Table 2): 2–3 ppm downfield shift for ^13^C^β^ and a 1–2 ppm upfield shift for ^13^C^α^ (Bienkiewicz & Lumb, 1999). Further analysis of other backbone assignment data for pS16M179 confirms that this new amide cross peak is preceded by a leucine residue and proceeded by a tyrosine residue, as expected from the primary amino acid sequence for amelogenin in this region, ‐LSY‐. Furthermore, the ^1^H‐^15^N HSQC spectrum of ^15^N‐labeled pS16M179 following incubation with lambda phosphatase, an enzyme that removes the phosphate group from phosphorylated serine, threonine, and tyrosine residues (Wera & Hemmings, 1995), was superimposable on the spectrum of non‐phosphorylated M179 (data not shown). In conclusion, the NMR data unambiguously shows that pSer has been incorporated into pS16M179 and the new cross peak in its ^1^H‐^15^N HSQC spectrum is pSer‐16.

### 2.3 Confirmation of pSer incorporation into pS16M179 by intact mass spectrometry

**FIGURE 4:**
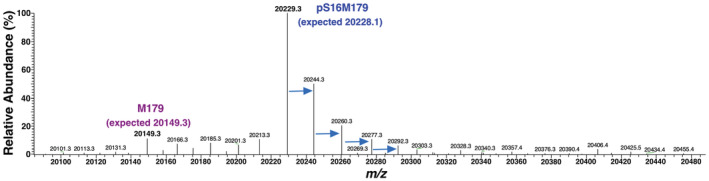
Deconvoluted monoisotopic intact mass spectrum for the unlabeled Celtone product (Celtone‐U) shown in the Figure 3 gels. The most abundant species at *m/z* = 20,229.3 corresponds to pS16M179 with an expected molecular weight of 20,228.1 Da. The four periodic species to the right of the major peak (highlighted with blue arrows) are protein oxidation products of increasing size, likely due to methionine oxidation. Out of the minor peaks, the only one consistent with the substitution of a native amino acid for pSer during translation is at *m/z* = 20,149.3 which corresponds to native M179 containing a serine with an expected molecular weight of 20,149.3 Da. The absence of peaks due to the substitution of any of the other 19 native amino acids suggests that pSer incorporation likely approaches 100% during translation and is dephosphorylated *in vivo* prior to purification

Analysis of mass spectral data to quantitate the fidelity of phosphoserine incorporation in NMR‐isotope labeled proteins is difficult because the isotope labeling is not 100%. Therefore, we used unlabeled Celtone complete media which is prepared identically to ^13^C‐, ^15^N‐labeled Celtone complete media except for ^13^C‐ and ^15^N‐enrichment. Moreover, as illustrated in Figure 1, the level of unphosphorylated amelogenin appears similar using unlabeled and ^13^C‐, ^15^N‐labeled Celtone complete media and Table 2 shows the yield of amelogenin is also similar. Figure 4 illustrates the deconvoluted monoisotopic mass spectrum for pS16M179 obtained from unlabeled Celtone complete media. The major peak at *m/z* 20,229.3 corresponds closely to the expected molecular weight of pS16M179 (20,228.1 Da). To the right of this major peak is a series of *m/z* peaks that increase by ~15 *m/z* units each. These are likely due to the oxidation of methionine residues which occurs spontaneously during the aqueous storage of protein under oxic conditions (Bettinger et al., 2020; Glaser & Li, 1974) (M179 contains nine methionine residues). Out of all the other minor peaks in the spectrum the only one corresponding to the substitution of a native amino acid for pSer is at *m/z* 20,149.3 which corresponds to a Ser residue. Because no other native amino acid substitutes for pSer during translation, this Ser likely is a result of the dephosphorylation of pSer inside *E. coli* cells prior to cell harvesting (centrifugation and then freezing at 193 K). It is unlikely due to dephosphorylation during the purification protocol because all the purifications were done similarly and included heating at 343 K under acidic conditions as the first step which will likely quickly denature any phosphatases. Note that this could also explain the higher level of non‐phosphorylated M179 using Celtone‐CDN (Figure 3) as these cells were harvested ~16 h after induction instead of ~4 h. Hence, the fidelity of pSer incorporation into M179 is likely near 100% but the final lower yields are due to dephosphorylation after translation. Assuming little difference in the ability to ionize M179 and pS16M179 in the gas phase during mass spectrometry data collection, the level of non‐phosphorylated amelogenin after purification was therefore ~5% using unlabeled Celtone media. Given that the Phos‐tag gel in Figure 1 suggests the level of unphosphorylated amelogenin is similar using either unlabeled or ^13^C‐, ^15^N‐labeled Celtone complete media, ~95% of the ^13^C‐, ^15^N‐labeled sample is phosphorylated at Ser‐16 which agrees with the ^1^H‐^15^N HSQC data. That is, there was no evidence for the Ser‐16 amide cross peak above the noise in the ^1^H‐^15^N HSQC spectrum for pS16M179 although it is evident above the noise in the spectrum from Celtone‐CDN where the level of non‐phosphorylated amelogenin is ~15% judging from the Phos‐tag gel in Figure 3 (data not shown).

While analysis of the two‐ and three‐dimensional NMR data convincingly shows that pS16M179 is phosphorylated at the correct position of the sequence, this was further corroborated by the analysis of fragmentation patterns for the major species in the mass spectrum of pS16M179 in Figure 4 as described in Figure S2.

### 2.4 Backbone amide chemical shift perturbations between pS16M179 and M179

The average combined perturbations between the ^1^H‐^15^N HSQC spectra of pS16M179 and M179 are shown in Figure 3b. The plot indicates that most of the chemical shift perturbations are less than 0.02 ppm (dashed line), a small value that is likely due to the resolution limits of the data (~6 Hz/point ^1^H, ~12 Hz/point ^15^N) or perhaps minor differences in the concentration of both samples. The most perturbed resonance is for Ser‐16/pSer‐16, with an average delta ppm of approximately 0.17 ppm. Around this major perturbation are a small cluster of resonances between Gly‐8 to Arg‐31, however, none of these perturbations are greater than 0.05 ppm. Previous site specific substitutions in M179, Thr‐21 to Ile, and Pro‐41 to Thr, generated larger chemical shift perturbations about the site of the substitution, up to ~0.3 ppm, under similar conditions of pH (Buchko et al., 2013). Hence, in 2% acetic acid at pH 2.8 where amelogenin is monomeric and intrinsically disordered, the substitution of pSer for Ser in pS16M179 had no significant structural effect on murine amelogenin (aside from Ser‐16/pSer‐16, there were no significant changes in any of the ^13^C^α^ and ^13^C^β^ chemical shifts for the other residues) nor significant perturbations to the local electronic environment.

### 2.5 ^31^P NMR spectra of pS16M179 in different quaternary states

**FIGURE 5:**
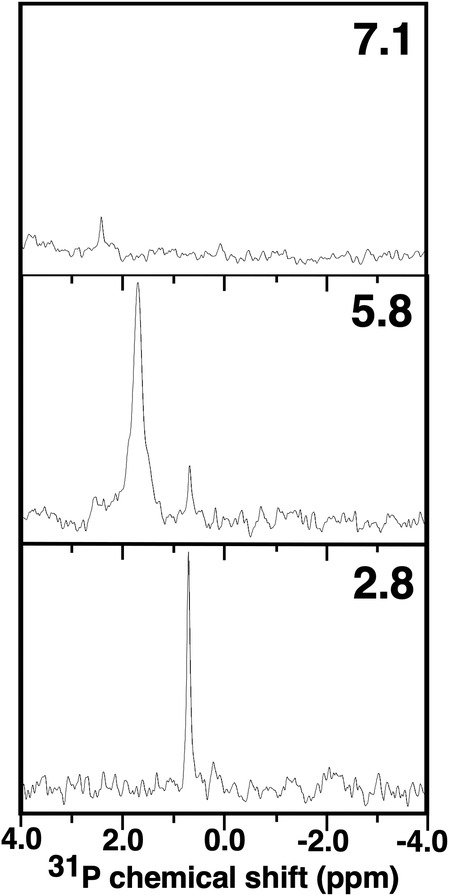
One‐dimensional ^31^P NMR spectra of pS16M179 at pH 7.1 (top), pH 5.8 (middle) and pH 2.8 (bottom) collected at 293 K and a ^1^H resonance frequency of 500 MHz. The top two spectra were for a 0.5 mM sample and the bottom spectrum is a 0.1 mM sample. The ^31^P spectra were referenced to 85% phosphoric acid (0.0 ppm)

At pH values above approximately seven amelogenin self‐assembles into nanospheres composed of 20–100 monomers (Du et al., 2005; Fang et al., 2011; Fincham et al., 1995). These structures are believed to be essential for proper enamel formation with the N‐terminal region identified as playing an essential role in amelogenesis (Paine & Snead, 1997). Single tryptophan fluorescence (constructs with only a single tryptophan, Trp‐25, Trp‐45, or Trp‐161) (Bromley et al., 2011) and NMR studies (Buchko et al., 2022) suggest the N‐terminal is buried within the nanospheres. This is corroborated by the absence of any significant ^31^P resonances in the one‐dimensional ^31^P NMR spectrum for 0.5 mM pS16M179 at pH 7.1 (Figure 5 (top)) after 20 h of data collection. The absence of a major ^31^P resonance is likely due to the same reasons amide resonances were not observed for the entire N‐terminal region of M179 in ^1^H‐^15^N HSQC spectra collected on ^2^H‐, ^13^C‐, ^15^N‐labeled M179 nanospheres (Buchko et al., 2022). In these ^1^H‐^15^N HSQC spectra the disappearance or intensity reduction of amide resonances were interpreted to reflect changes in dynamics (intermediate ms‐μs motion) and/or multiple chemical environments (heterogenous interfaces) of amide nuclei at protein–protein interfaces (Buchko, Tarasevich, et al., 2008). While the absence of a major ^31^P resonance is consistent with the N‐terminal region around pSer‐16 being buried and at protein‐protein interfaces, it is not buried 100% of the time. The observation that lambda phosphatase was able to remove the phosphate group from pS16M179 in buffers favorable for nanosphere formation (pH 7.1 and pH 7.8), as shown in Figure 1, is in agreement with previous studies suggesting monomers (Fincham et al., 1994; Limeback & Simic, 1990) or small oligomers (Tao et al., 2015) are in dynamic equilibrium with nanospheres. Indeed, there is a minor, relatively sharp resonance at 2.2 ppm which may represent the pSer‐16 resonance in monomeric amelogenin that was observed at 1.2 ppm at pH 2.8 (Table 1 and Figure 4). This is consistent with the pH dependence of the ^31^P chemical shift over the pH range of 2–9, moving downfield with increasing pH (Bienkiewicz & Lumb, 1999; Hoffmann et al., 1994).

An attempt was made to follow the lambda phosphatase dephosphorylation of pS16M179 by ^31^P NMR spectrometry using an unlabeled sample (0.5 mM). As just noted, no major ^31^P resonance was observed in the one‐dimensional spectrum before the additional of the phosphatase (Figure 4, top), but, it was anticipated that it would be possible to follow the reaction by the appearance of a signal for the free phosphate. Although lambda phosphatase had converted pS16M179 completely to M179, as shown in the Phos‐tag gel in Figure 1, no signal was observed for the free phosphate in the one‐dimensional ^31^P NMR spectrum. This suggests that after cleavage the free phosphate bound to amelogenin nanospheres.

While the N‐termini of amelogenin appears to be largely buried in nanospheres at pH values above seven, this region appears less buried in the transition from monomers to oligomers as the pH is increased from 3 to ~6.8 (Bromley et al., 2011). A chemical shift perturbation study as a function of pH enabled the tracking of the protein–protein interface at the residue level and showed that at pH 6.6 Ser‐16 and a region around it, Ser‐9 to Leu‐20, was not intimately part of an oligomer interface (Buchko et al., 2022). This is corroborated in Figure 5 (middle), the one dimensional ^31^P NMR spectrum for 0.5 mM pS16M179 at pH 5.8, where a major resonance is observed at 1.7 ppm. Not only is this resonance shifted relative to the single resonance observed at pH 2.8 (Figure 5, bottom), it is also broader, a characteristic of a species with a larger molecular weight. Note that a second minor resonance at 0.7 ppm, a similar chemical shift observed for pS16M179 at pH 2.8, is also present. While it would be tempting to suggest this is the chemical shift for monomeric pS16M179, the pKa of the phosphoryl group of pSer in peptides is 5.96 and the ^31^P chemical shift is pH dependent over the pH range of 2–9, moving downfield with increasing pH (Bienkiewicz & Lumb, 1999; Hoffmann et al., 1994). Hence, the reason for the two resonances is unclear especially since only a single resonance was observed for the amide chemical shift of S16 in M179 at pH 6.6 (Buchko et al., 2022). Efforts are in progress to determine the reason for the two ^31^P resonances for pS16M179, including the collection of NMR backbone assignment data on ^2^H‐, ^13^C‐, ^15^N‐labeled pS16M179 at pH 5.8.

## 3 CONCLUSIONS

Using third generation genetic code expansion protocols we have shown it is possible to express and purify recombinant, unlabeled, bacterial murine amelogenin in high yields with a level of pSer incorporation at the native Ser‐16 position approaching 95% (the fidelity of incorporation is likely near 100% but some *in vivo* dephosphorylation after translation is suspected). Using enriched, ^13^C‐ and ^15^N‐labeled commercial media it is possible to uniformly ^13^C‐, ^15^N‐label amelogenin for NMR studies with the same level of pSer incorporation. Sacrificing yield (~50% less, Table 1) and pSer incorporation levels (~85%, Figure 2), amelogenin may be perdeuterated for NMR experiments designed to assist studies of large amelogenin quaternary structures (Buchko et al., 2022) using enriched, ^2^H‐, ^13^C‐, and ^15^N‐labeled commercial media. The approaches for NMR labeling all use IPTG to induce protein expression. For experiments not requiring NMR isotope labeling of amelogenin and only pSer incorporation, ZY‐ or LB‐media may be used instead of unlabeled enriched commercial media (Celtone or BioXpress) with IPTG induction of protein expression to obtain various levels (~85%–95%) of pSer incorporation (as estimated by the Phos‐tag gel in Figure 1). While our final levels of pSer incorporation were high, certainly above levels where they would not complicate interpretation of NMR data, they were not 100%. For pS16M179 experiments requiring near 100% levels of pSer incorporation, we suspect that it may be possible to coax the *E. coli* closer toward 100% by optimizing growth conditions yet further through experimenting with growth parameters such as: optical density at induction, the concentration of IPTG used at induction, the temperature, and perhaps most importantly, the length of induction before cell harvesting as mass spectrometry suggests non‐phosphorylated M179 is largely due to dephosphorylation of pS16M179 in *E. coli* after translation.

The observation of two resonances in the one‐dimensional ^31^P NMR spectrum of pS16M179 at pH 5.8 is just one example of the new insights to be discovered regarding the biophysical properties of amelogenin provided by the ability to site‐specifically incorporate pSer into the primary amino acid sequence of amelogenin. Moreover, because the major NMR‐detectable natural isotope of phosphorus, phosphorus‐31, is 100% abundant, unlabeled pS16M179 contains a label near the N‐terminal that can be readily followed by ^31^P NMR spectroscopy. In conclusion, the high‐yield ^13^C‐, ^15^N‐labeling of amelogenin with high levels of phosphoserine incorporation shown here will not only accelerate biomineralization research to understand amelogenesis, but, trigger the expanded use of the third generation genetic code expansion protocols to introduce phosphorylated amino acids into other proteins to better understand the structural and functional consequences of the most common posttranslational modification cells use to regulate protein function.

## 4 METHODS

### 4.1 Cloning, expression, and purification

DNA encoding the primary amino acid sequence for native murine amelogenin (Simmer et al., 1994) was codon optimized with a TAG codon inserted for Ser‐16, synthesized by IDT (Corvaville, Iowa), and inserted into a pRBC plasmid (Addgene # 173897) containing an ampicillin resistance gene (Zhu et al., 2019) using PPY‐based SLiCE techniques (Zhang et al., 2012). The plasmid containing the machinery for pSer incorporation, pKW2‐EPSep, contained a chloramphenicol resistance gene and was a gift from Jason Chin. Initial evaluations for expressing phosphorylated sfGFP (sfGFP‐150TAG) in the release Factor‐1 (RF1) deficient *E. coli* B95(DE3) ΔA ΔfabR ΔserB strain in media amenable to isotopic enrichment (Figure S1) were performed as previously described (Vesely et al., 2022). Subsequent protein expression was achieved following a 4 day protocol (Zhu et al., 2019). *Day 1*. Both plasmids were used to simultaneously transform a competent, Release Factor‐1 (RF1) deficient, *E. coli* B95(DE3) ΔA ΔfabR ΔserB strain using a standard heat shock method (30 min ice; 315 K, 45 s; 2 min ice). After 1 ml addition of SOC media, the cells were allowed to recover with shaking for 2 h at 310 K. All the cells were streaked on ampicillin (25 μg/ml) and chloramphenicol (7 μg/ml) LB‐agar plates by gently spinning (3 min @ 3000 g), removing top 900 μl, and resuspending the pellet in the remaining 100 μl of media. *Day 2*. Following overnight incubation at 310 K, the plates were transferred to a 277 K fridge for the day. Late afternoon, a 25 ml starter culture was prepared starting with approximately 10 colonies using a buffered, glucose‐rich, non‐autoinducing medium (ZY‐NIM) (Zhu et al., 2019) containing ampicillin (50 μg/ml) and chloramphenicol (14 μg/ml). The culture was shaken at 310 K overnight in a baffled 250 ml flask at ~200 rpm. *Day 3*. The optical density of the starter culture at 600 nm was measured to ensure it had grown to saturation (OD_600_ = 3–8). Upon reaching growth saturation, 1 ml of this culture was added to 100 ml of ^13^C‐, ^15^N‐labeled Celtone complete media (Cambridge Isotopes Laboratory, Tewksbury, MA) in a 500 ml baffled flask and incubated at 310 K with shaking (200 rpm). At an OD_600_ of ~0.8 protein expression was induced by the addition of isopropyl β‐D‐1‐thiogalactopyranoside (IPTG; 0.026 mg/ml) and incubation continued under the same conditions. *Day 4*. After approximately 4 h the cells (OD_600_ ~ 1.7) were harvested by mild centrifugation and frozen at 193 K.

In addition to using 100 ml of ^13^C‐, ^15^N‐labeled Celtone complete media on *Day 3* in the protein expression protocol, other commercial media were also tested including ^2^H‐, ^13^C‐, ^15^N‐labeled and unlabeled Celtone complete media, ^15^N‐labeled BioExpress complete media (10× concentrate; Cambridge Isotopes Laboratory, Tewksbury, MA), and ^13^C‐, ^15^N‐labeled and unlabeled Celtone powder (Cambridge Isotopes Laboratory, Tewksbury, MA) (0.2% w/v in Miller minimal media). Non‐commercial media tested were LB and ZY using both IPTG induction (0.026 mg/ml at OD_600_ of ~0.8) and autoinduction (Studier, 2005). Cells were harvest ~4 h after induction with IPTG except with ^2^H‐, ^13^C‐, ^15^N‐labeled Celtone complete media where harvesting occurred ~16 h later. Harvesting of autoinduction cultures occurred the following morning.

Amelogenin was purified following a previously published protocol (Buchko & Shaw, 2015). For 100 ml of cell culture, 20 ml of 2% acetic acid was added to the frozen pellet in a 50 ml centrifuge tube. The solution was incubated at ~343 K for ~15 min in a water bath (with a couple manual hard shakes in between), sonicated for 2 min (5 s on, 2 s off, 25% level) on a Qsonica GEX750 sonicator (Newton, CT), incubated for a further 15 minutes in the water bath followed by another 2 min of sonication under the same conditions. The entire solution was placed in a 3.5 kDa molecular weight cutoff dialysis tubing (Fisher Scientific, Waltham, MA) and dialyzed in 5 l of 2% aqueous acetic acid twice (over 24–36 h) at 277 K. The insoluble cell debris was removed by centrifugation for 45 min at 17,500 rpm in a Beckman J‐20 rotor in a Beckman Avanti J‐25 centrifuge (Fullerton, CA). The supernatant was applied to a Resource‐RPC 3 ml column (GE Healthcare, Uppsala, Sweden) in 5–10 ml volumes and the following linear purification gradient applied: Buffer A = 100% water in 0.005% TFA, Buffer B = 70% aqueous acetonitrile in 0.005% TFA; flow rate = 3 ml/min; Step 1–100% Buffer A 5 CV, Step 2––linear gradient 0–100% Buffer B. The fractions containing amelogenin eluting at ~55% Buffer B were pooled, frozen at 193 K, and lyophilized. To assess the purity of the products and the extent of pSer incorporation, regular 4%–12% TrisHCl SDS‐PAGE gels and 12.5% SuperSep Phos‐tag SDS‐PAGE gels (199–18,011, FUJIFILM Wako Chemicals USA, Richmond, VA), respectively, were run. The Phos‐tag gel was run following the manufacturer's instructions at 120 V constant voltage for 2 h and stained with GelCode Blue Safe Protein Stain (ThermoFisher Scientific, Rockford, IL). The yields of purified amelogenin were as measured using absorption at 280 nm, a calculated molar extinction coefficient of 25,440 M^−1^ cm^−1^, and a molecular weight of 20,160 Da. Note that amelogenin yields (total protein) were ~50% greater if 6 M guanidinium hydrochloride was used instead of 2% acetic acid in the cell lysis step (followed by centrifugation prior to dialysis) (Buchko & Shaw, 2015). However, this method resulted in some additional impurities that co‐eluted with amelogenin on the reverse phase column, and therefore, this method was avoided in these studies.

### 4.2 NMR spectroscopy

Lyophilized pS16M179 was resuspended in an NMR buffer consisting of 2% CD_3_CO_2_D, 7% D_2_O/93% H_2_0, pH 2.8, to a concentration of 0.3 mM (measured using a calculated ε of 25,440 M^−1^ cm^−1^). All the NMR data was collected at 293 K on four‐channel Varian‐600 NMR spectrometers equipped with triple‐resonance probes and pulse field gradients to verify chemical shift assignment and the presence of pSer in the sequence. This included two‐ and three‐dimensional ^1^H‐^15^N HSQC, ^1^H‐^13^C HSQC, HNCA, HNCO, HNCACB, HNCOCACB, and HNN experiments (Varian Biopack pulse programs). One‐dimensional ^31^P NMR spectra were collected for pS16M179 and pSer on a Agilient‐DD2 NMR spectrometer operating at a ^1^H proton resonance frequency of 499.92 MHz with proton decoupling and referenced to 85% phosphoric acid (0.0 ppm). The O‐phospho‐L‐serine (P0878) was purchased from Sigma‐Aldrich (St. Louis, MO).

### 4.3 Intact protein mass spectrometry

The protein solution at 1.2 μg/μl was diluted in water to 24 ng/μl. Ten μl was loaded (~240 ng) onto a Waters NanoAcquity LC with online desalting. Reversed phase separation was carried out on an in‐house packed C2 column (100 μm i.d., ~50 cm long, packing material SMTC2MEB2–3‐300 from Separation Methods Technologies, Newark, DE). Mobile phases were 0.2% formic acid in water (A) and 0.2% formic acid in acetonitrile (B). A linear gradient with a flow rate of 0.3 μl/min was run from 15% to 50% mobile phase B over 30 min. The protein eluted between 30%–40% mobile phase B. Mass spectrometry data were collected on a Thermo Orbitrap Exploris. The mass spectrum (120 k resolution, 5 microscans) across the elution window were summed, and deconvoluted with FreeStyle (v1.5, ThermoScientific) for estimating the relative abundance of the phosphorylated protein.

### 4.4 Lambda phosphatase reaction

Approximately 0.8 mg of lyophilized, ^15^N‐labeled pS16M179 was resuspended in 250 μl of water and allowed to sit at room temperature for 24 h. This solution was added to a 250 μl solution containing 50 mM TrisHCl, pH 8.5, while gently vortexing. Fifty μl of 10× Reaction Buffer (500 mM HEPES, 1 mM EGTA, 50 mM dithiothreiotol, 0.1% BRIJ 35), 50 μl of 10× MgCl_2_ (20 mM), and 5 μl of lambda phosphatase (400 units/uL, Santa Cruz Biotechnology, Dallas, TX) was added to this solution that had a final pH of 7.8. The reaction solution was incubated overnight at 303 K. Prior to purification by reverse phase chromatography as described above, an equal volume of reverse phase Buffer A was added to the solution and the pH adjusted to ~3 with 1 μl additions of TFA. A similar experiment was performed on a 0.5 mM sample of unlabeled pS16M179 in a Shigemi NMR tube (260 μl) under the same buffer conditions except the pH was adjusted to 7.1 prior to the addition of 5 μl of lambda phosphatase.

## AUTHOR CONTRIBUTIONS

**Garry W. Buchko:** Conceptualization (lead); data curation (lead); funding acquisition (equal); investigation (lead);  methodology (equal); resources (equal); supervision (lead); visualization (lead); writing ‐  original draft (lead); writing – review and editing (lead). **Mowei Zhou:** Investigation (supporting). **Cat Hoang Vesely:** Investigation (supporting); methodology (supporting). **Jinhui Tao:** Funding acquisition (equal); resources (equal). **Wendy J. Shaw:** Funding acquisition (lead); project administration (lead). **Ryan A. Mehl:** Conceptualization (supporting); investigation (supporting); methodology (equal); resources (supporting); writing – review and editing (supporting). **Richard B. Cooley:** Conceptualization (supporting); investigation (supporting); methodology (equal); resources (supporting); supervision (supporting); visualization (supporting); writing – review and editing (supporting).

## Supporting information


**Supplemental Figure 1.** (a) Final optical density at 600 nM (OD_600_) of cultures encoding phosphoserine into a sfGFP‐150TAG reporter gene (super‐folder green fluorescent protein with an amber (TAG) stop codon for phosphoserine at residue position 150) grown in high and low density methods, using RF1‐containing BL21(DE3) ΔserB or RF‐1 deficient B95(DE3) ΔA ΔfabR ΔserB, at 25°C and 37°C, as indicated. (b) Fluorescence of the same cultures shown in panel (a). Only when the 150TAG codon in sfGFP is suppressed (either by near‐cognate suppression or pSer incorporation) is full‐length sfGFP produced, causing cells to fluoresce. Culture fluorescence therefore reflects the amount of TAG codon suppressed and full‐length sfGFP made. (c) SDS‐PAGE (top) and Phos‐tag (bottom) electrophoresis of proteins purified from cultures shown in panels (a, b). The Phos‐tag gel behaves like an SDS‐PAGE gel except the former contains a di‐nuclear metal complex with affinity for phosphate groups that retards migration of phosphorylated protein in proportion to the number of phosphorylated sites. When sfGFP‐150TAG was expressed in B95(DE3) ΔA ΔfabR ΔserB at low density and 37°C, approximately 80%–90% of the protein was phosphorylated.
**Supplemental Figure 2.** (a) Fragmentation spectrum of the 20,229 Da species, showing good matches of major fragments corresponding to the phosphorylation at Ser‐16 (numbered 15 in data due to the absence of Met‐1). The b‐ions (larger than b15) contained the intact phosphate group with +79.966 Da. (b) Expanded view showing several continuous signature b‐ions with neutral loss, which are highlighted in yellow boxes. These b‐ions lost the phosphate group and showed water loss on Ser‐16 (−18.01 Da), which is common for collisional activation of phosphorylated proteins and peptides. The b15 fragment with neutral loss help localize the phosphorylation on Ser‐16. (c) Protein coverage map matching to neutral loss at S16 (−18.01 Da). The blue labels show the b‐ion coverage from the N‐terminal and the red labels show the y‐ion coverage from the C‐terminal. The overall coverage is 27.5%.

## Data Availability

Data available upon request from the authors.
