# Supplementary material for: High‐yield recombinant bacterial expression of 13C‐, 15N‐labeled, serine‐16 phosphorylated, murine amelogenin using a modified third generation genetic code expansion protocol
Source: Protein Sci. 2023 Feb 1;32(2):e4560. doi: 10.1002/pro.4560 (PMC9850436; doi:10.1002/pro.4560)
Supplement: Supplementary file 1 — Supplemental Figure 1. (a) Final optical density at 600 nM (OD600) of cultures encoding phosphoserine into a sfGFP‐150TAG reporter gene (super‐folder green fluorescent protein with an amber (TAG) stop codon for phosphoserine at residue position 150) grown in high and low density methods, using RF1‐containing BL21(DE3) ΔserB or RF‐1 deficient B95(DE3) ΔA ΔfabR ΔserB, at 25°C and 37°C, as indicated. (b) Fluorescence of the same cultures shown in panel (a). Only when the 150TAG codon in sfGFP is suppressed (either by near‐cognate suppression or pSer incorporation) is full‐length sfGFP produced, causing cells to fluoresce. Culture fluorescence therefore reflects the amount of TAG codon suppressed and full‐length sfGFP made. (c) SDS‐PAGE (top) and Phos‐tag (bottom) electrophoresis of proteins purified from cultures shown in panels (a, b). The Phos‐tag gel behaves like an SDS‐PAGE gel except the former contains a di‐nuclear metal complex with affinity for phosphate groups that retards migration of phosphorylated protein in proportion to the number of phosphorylated sites. When sfGFP‐150TAG was expressed in B95(DE3) ΔA ΔfabR ΔserB at low density and 37°C, approximately 80%–90% of the protein was phosphorylated. Supplemental Figure 2. (a) Fragmentation spectrum of the 20,229 Da species, showing good matches of major fragments corresponding to the phosphorylation at Ser‐16 (numbered 15 in data due to the absence of Met‐1). The b‐ions (larger than b15) contained the intact phosphate group with +79.966 Da. (b) Expanded view showing several continuous signature b‐ions with neutral loss, which are highlighted in yellow boxes. These b‐ions lost the phosphate group and showed water loss on Ser‐16 (−18.01 Da), which is common for collisional activation of phosphorylated proteins and peptides. The b15 fragment with neutral loss help localize the phosphorylation on Ser‐16. (c) Protein coverage map matching to neutral loss at S16 (−18.01 Da). The blue labels show the b‐ion coverage fr [file PRO-32-e4560-s001.docx]

**SUPPLEMENTAL INFORMATION**

**High-yield recombinant bacterial expression of ^13^C-, ^15^N-labelled, serine-16 phosphorylated, murine amelogenin using a modified third generation genetic code expansion protocol**

Garry W. Buchko^1,2,^*, Mowei Zhou^1^, Cat Hoang Vesely^3^, Jinhui Tao^4^, Wendy J. Shaw^4^, Ryan A. Mehl^3^, and Richard B. Cooley^3,*^

^1^Earth and Biological Sciences Directorate, Pacific Northwest National Laboratory, Richland, Washington.

^2^School of Molecular Biosciences, Washington State University, Pullman, Washington

^3^Oregon State University, Department of Biochemistry and Biophysics, 2011 Agricultural and Life Sciences, Corvallis, Oregon

^4^Physical and Computational Sciences Directorate, Pacific Northwest National Laboratory, Richland, Washington

*Corresponding authors

E-mail addresses: [garry.buchko@pnnl.gov](mailto:garry.buchko@pnnl.gov); rick.cooley@oregonstate.edu

**Mass spectral verification of a pSer at position 16 in pS16M179**

Data dependent MS-MS data collection (120k resolution, 2 microscans) was performed using higher-energy collisional dissociation (HCD) with 35% energy. The MD-MS data were first analyzed using TopPIC^1^ to identify best spectra for the target proteins, then selected spectra were manually examined and annotated to confirm the phosphorylation site using LcMsSpectator.^2^ Mass tolerance was 15 ppm.


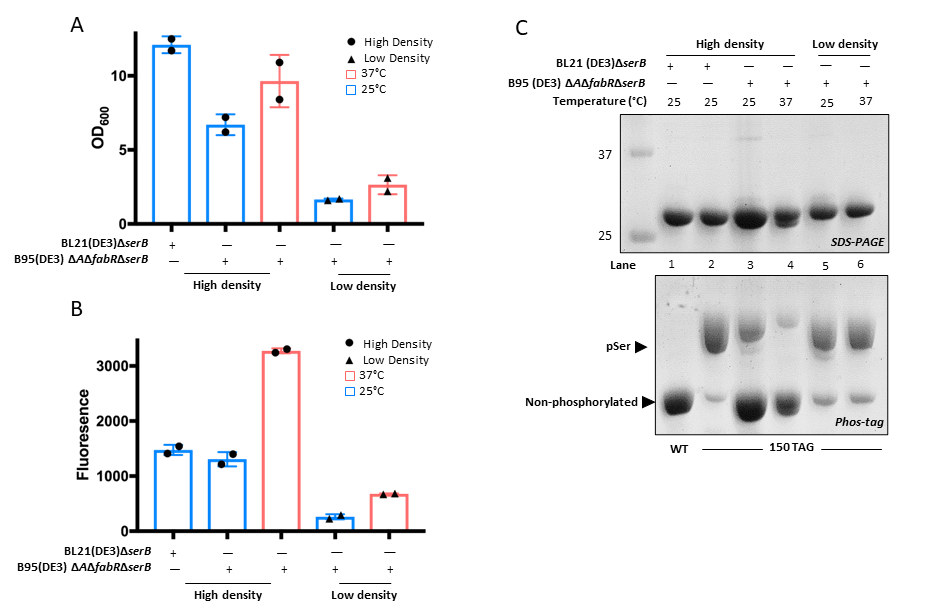


**Supplemental Figure 1.** (**A**) Final optical density at 600 nM (OD_600_) of cultures encoding phosphoserine into a sfGFP-150TAG reporter gene (super-folder green fluorescent protein with an amber (TAG) stop codon for phosphoserine at residue position 150) grown in high and low density methods,^3^ using RF1-containing BL21(DE3) ΔserB or RF-1 deficient B95(DE3) ΔA ΔfabR ΔserB, at 25 and 37 ^o^C, as indicated. (**B**) Fluorescence of the same cultures shown in panel (**A**). Only when the 150TAG codon in sfGFP is suppressed (either by near-cognate suppression or pSer incorporation) is full-length sfGFP produced, causing cells to fluoresce. Culture fluorescence therefore reflects the amount of TAG codon suppressed and full-length sfGFP made. (**C**) SDS-PAGE (top) and Phos-tag (bottom) electrophoresis of proteins purified from cultures shown in panels (**A**) and (**B**). The Phos-tag gel behaves like an SDS-PAGE gel except the former contains a di-nuclear metal complex with affinity for phosphate groups that retards migration of phosphorylated protein in proportion to the number of phosphorylated sites. When sfGFP-150TAG was expressed in B95(DE3) ΔA ΔfabR ΔserB at low density and 37 ^o^C, approximately 80-90% of the protein was phosphorylated.


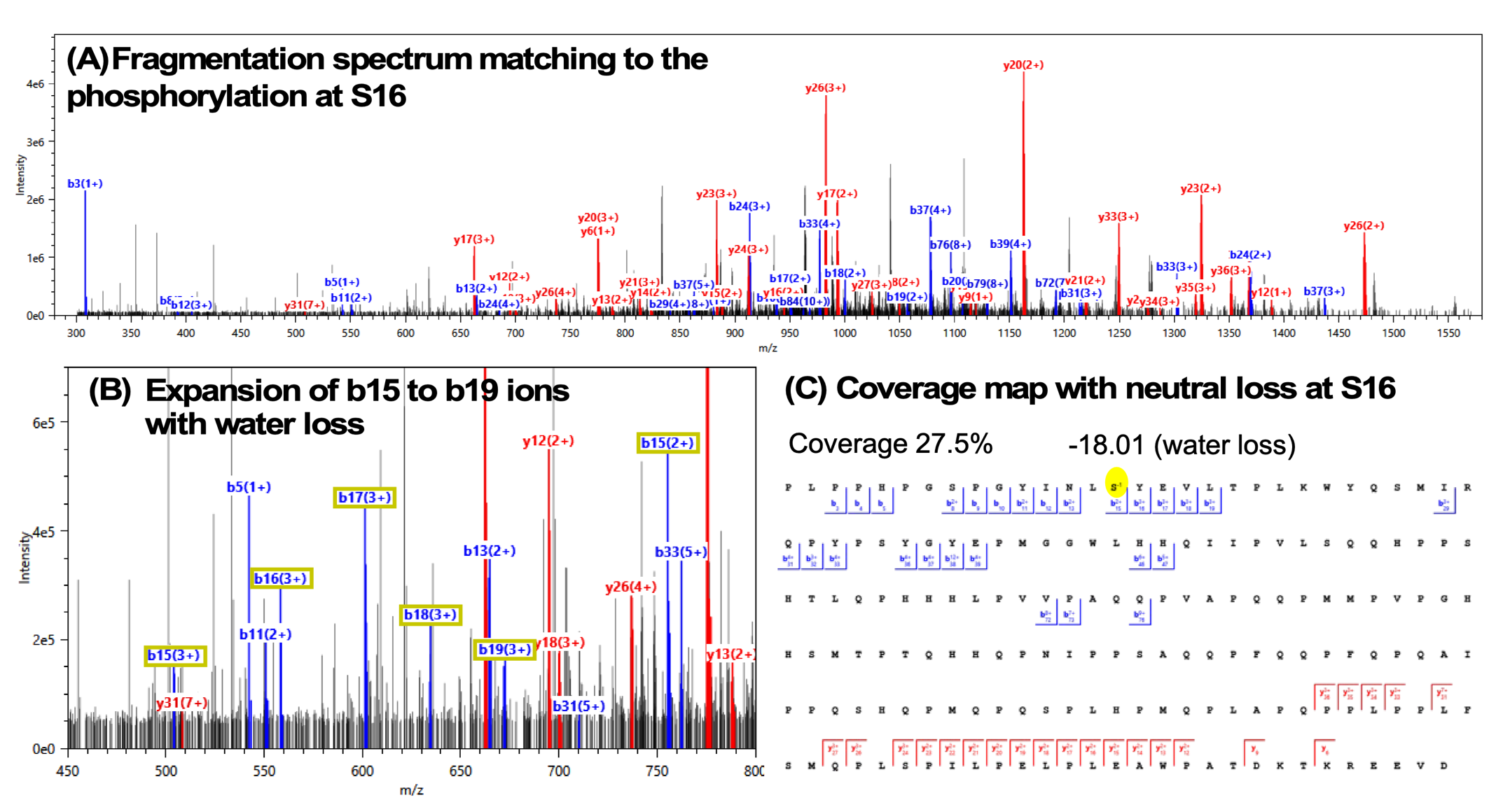


**Supplemental Figure 2.** (**A**) Fragmentation spectrum of the 20229 Da species, showing good matches of major fragments corresponding to the phosphorylation at Ser-16 (numbered 15 in data due to the absence of Met-1. The b-ions (larger than b15) contained the intact phosphate group with +79.966 Da. (**B**) Expanded view showing several continuous signature b-ions with neutral loss, which are highlighted in yellow boxes. These b-ions lost the phosphate group and showed water loss on Ser-16 (-18.01 Da), which is common for collisional activation of phosphorylated proteins and peptides. The b15 fragment with neutral loss help localize the phosphorylation on Ser-16. (**C**) Protein coverage map matching to neutral loss at S16 (-18.01 Da). The blue labels show the b-ion coverage from the N-terminal and the red labels show the y-ion coverage from the C-terminal. The overall coverage is 27.5%.

**References**

1. Kou Q, Xun LK, Liu XW. TopPIC: a software tool for top-down mass spectrometry-based proteoform identification and characterization. Bioinformatics. 2016;32:3495-3497.

2. Park J, Piehowski PD, Wilkins C, Zhou M, Mendoza J, Fujimoto GM, Gibbons BC, Shaw JB, Shen Y, Shukla AK, Moore RJ, Liu T, Petyuk VA, Tolic N, Pasa-Tolic L, Smith RD, Payne SH, Kim S. Author Correction: Informed-Proteomics: open-source software package for top-down proteomics. Nat Methods. 2018;15:554.

3. Vesely CH, Reardon PN, Yu Z, Barber E, Mehl RA, Cooley RB. Accessing isotopically labeled proteins containing genetically encoded phosphoserine for NMR with optimized expression conditions. J Biol Chem. 2022;298:102613.
